# Expression of circulating micro-RNAs in hypertensive patients with left ventricular hypertrophy

**DOI:** 10.1186/1471-2458-12-S2-A34

**Published:** 2012-11-27

**Authors:** Soh Zi Ling, Chee Kok Han, Wong Chew Ming, Wang Chee Woon

**Affiliations:** 1 Department of Medicine, Faculty of Medicine, University of Malaya, 50603 Kuala Lumpur, Malaysia; 2 Department of Biochemistry, MAHSA University College, Jalan Elmu off Jalan Universiti, 59100 Kuala Lumpur, Malaysia

## Background

Left ventricular hypertrophy (LVH) is a common presentation among hypertensive patients. Micro-RNAs (miRNAs) have been proven to show reproducible dysregulations in hypertrophied cardiac tissues. Three miRNAs, miR-133, miR-155, and miR-195 were used in the study to elucidate the miRNA expression anomaly in heart diseases. MiR-133 and miR-195 have been reported as two highly expressed miRNAs in normal mouse heart tissue. The aim of this study is to observe the relationship of the levels of circulating miR-133, miR-155 and miR-195, with the phenotype of cardiac hypertrophy among LVH patients and normal controls.

## Materials and methods

Forty-four hypertensive patients and 26 unrelated healthy individuals were selected and their peripheral blood were collected through venipuncture. Total RNA was then extracted and reverse transcribed into cDNA. The cDNA templates were subjected to quantitative real time polymerase chain reaction. The expression levels of miR-133, miR-155, and miR-195 were compared between the patient and the control groups. Echocardiograms were also obtained from all subjects to evaluate the condition of the heart.

## Results

The delta CT means of miR-133, miR-155 and miR-195 were 17.427, 13.343, and 9.406, respectively for the control group. However, the delta CT means of miR-133, miR-155 and miR-195 for the patient group were 18.078, 13.8427, and 10.2295, respectively. The expression levels of miR-133, miR-155 and miR-195 were found to be significantly down-regulated in patients with p values of 0.0306, 0.03567, and 0.0105, respectively.

## Conclusions

Our data show that the down-regulated expression of miR-133 and miR-155 in the presence of LVH reported in previous tissue and in vivo studies is also reflected in the circulating levels. Interestingly, the pattern of dysregulation observed in circulatory miR-195 expression level in hypertrophic patients disagrees with published in vivo studies. A larger sample size could prove useful in the validation of miR-133, miR-155 and miR-195 as biomarkers of LVH development. Further studies to illuminate the targets and mechanisms involved would be beneficial in the efforts to reverse this debilitating condition.

